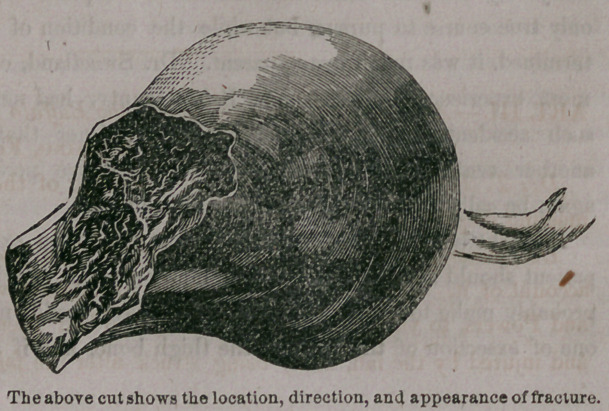# Abstract of the Proceedings of the Buffalo Medical Association

**Published:** 1864-03

**Authors:** J. F. Miner

**Affiliations:** Secretary


ART. III.—*Abstract of the Proceedings of the Buffalo Medical Association.*

TUESDAY EVENING, February 2d, 1864.

*Dr. T. T. Lockwood* in the Chair. Reading of the minutes of the last meeting dispensed with.

*Dr. Miner* presented the head of a thigh bone, and gave the following account of it. Was called yesterday in consultation with Drs. Sweetland and Powers, to visit Mr. Cash of Evans, who had been thrown off his sled and injured by the fall, or by being struck after the fall, or both. He was carried home and Dr. Sweetland was immediately in attendance, who on discovering the nature of the injury associated with himself Dr. Powers.—It was now discovered that they had *fracture and dislocation* of the thigh bone, the upper portion or rather the head resting in the inguinal region, or immediately below it, the femoral artery passing over the bone and imparting an impulse to the tumor not at first satisfactorily accounted for. The remarkable injury, and the peculiar condition of the parts induced hesitancy as to the proper course to pursue, and the leg was placed over a pillow in flexed position, to relieve the tension, and in this condition awaited his arrival. Careful examination confirmed the diagnosis, and no doubt was entertained that the head of the thigh bone had been driven from the acetabulum and separated at the neck, from the shaft of the bone. A condition of things so rare and so inexplicable, very naturally led to some doubts and anxieties, but as it was established that fractured bones should be replaced, effort was made while the patient was fully under the influence of chloroform, to replace the dislocated portion. This effort, though entirely futile, so far as accomplishing this object, was yet productive of benefit, making more apparent the complete isolation of the fragment, and the utter impossibility of replacing it. It had also dislodged it from under the femoral vessels, and rendered it movable beneath the integuments outside the muscles and fascia of the thigh. Its removal was now suggested to the attending physicians as the only rational plan of procedure. It was obvious it could not be replaced if desirable, and though it was impossible to know how complete was the isolation, yet it appeared probable—certain almost, that it had no attachments for support, and was completely a foreign substance. Upon suggestion, it was immediately approved, and its removal unanimously agreed to, as the only safe and proper course to pursue, not however without the conviction that to make incision down upon the displace head of a thigh bone, with the view of removal, because it was separated from the shaft and displaced, was a new and unheard of operation. Incision was however unhesitatingly made, and the head and neck as here seen,

The above cut shows the location, direction, and appearance of fracture.

was extracted. The ligamentum teres had been broken, the capsular ligament ruptured, the neck of the bone fractured within the capsular ligament, and the bare specimen exactly as here presented, without any attachments was taken from underneath the integument. The specimen has been presented not that it is unlike the head of other thigh bones, but because it is so remarkable a relic.

It was not presumed, but that in some of the terrible contusions of gunshot or railroad accidents somewhat similar injuries might be sustained by the neck and head of the thigh bone; but that such a condition should be found in connection with no outward contusion or laceration, nothing but simple fracture so far as the shaft of the femur was involved, was regarded as the rarest injury in the world, and one which perhaps had not its parallel recorded.

What forces could have produced such injury is not readily apparent, but it appears that the dislocation must have taken place first, the head of the bone being protruded through the capsule, muscles and fascia, and subsequently another blow have produced the fracture; when the shaft was again drawn back to its natural position, leaving the head entirely disconnected—a foreign substance so far as nutrition is concerned. This then was accidental exsection of the head of the thigh bone; and from what is known of the results of severe injuries of the femur, especially of the head, and of the hip joint, they had nothing to promise the friends of this patient; it would appear altogether probable that death would soon follow so severe injury and such operation. He was however left perfectly comfortable, as much so as is usual after simple fracture of the neck of the thigh bone. The usual dressings for such accident were applied without attempting very great extension; the shortening was slight, and the appearance every way natural. After the removal, it was plain enough that it was the only true course to pursue, but while the condition of the bone was undetermined, it was much less apparent. Dr. Sweetland, one of the oldest and most experienced physicians of Erie County, had never seen or heard of such accident, and it was believed by Dr. Miner that if he should live another century and practice surgery the entire period, he would never again be called to attend a similar injury.

It might be interesting to inquire what was to be expected in case this patient should survive the shock of injury—what provision would nature probably make to supply the loss? The case could not be greatly unlike one of exsection of the head of the thigh bone, and if a favorable termination should take place, it could not but afford as satisfactory result, and on some accounts much more was to be expected. The angle formed by the neck was still left, constituting a base upon which nature could build a useful and firm head for articulation with the acetabulum; and bones which are fractured without being crushed and comminuted, take on healthy granulation sooner, and heal over more rapidly and more safely, than when divided by the saw. Recovery from so severe an injury, involving the hip joint, appears altogether improbable, and yet perhaps it is not impossible.*

*Dr. Boardman* would like to inquire as to the protection of vaccination. Had re-vaccinated cases two weeks after vaccination without effect, but subsequently found that re-vaccination took effect. Did not understand why the second re-vaccination should take effect while the first did not, which was made two weeks before, and with reliable virus.

*Dr. Cronyn* had noticed the same thing as related by Dr. Boardman, and related cases illustrative of it. Re-vacninated on Sixth street four children, three of them failed to produce effect, while the other one worked very well. The mother was not satisfied with the virus where it did not work, and in a few weeks he vaccinated them again, when it took finely.—The virus in both cases was fresh and reliable.

*Dr. Rochester* was glad the discussion had been commenced, since the importance of vaccination could not be over-estimated, and everything connected with it was of interest. Spoke of the uncertainty of vaccination, and of the common causes of its failure; impurity or other imperfections in the virus being the most operative and apparent of any. As he had often before remarked in the Society, he greatly preferred the lymph. When the scale is used we always introduce pus, though vaccine lymph may be incorporated with it.

Said that it was a common occurrence to vaccinate with the crust with a perfect failure, when perhaps vaccination with the same virus two weeks later would take, and with it the old virus would also be lighted up which had laid dormant for many days, and would now produce true vaccine vesicle. Thought there was now an epidemic pre-disposition to small pox, and that possibly this might account for the tendency in vaccination to take effect. Related cases which could not previously be made to take, which have recently taken and passed through the stages beautifully. Has also

* Recent report from attending physician, Dr. Powers, assures us that the case is progressing favorably. Mr. Cash's son called upon us yesterday, February 17th, twenty days after the injury, and informs us that his father is free from pain or other inconvenience, eating naturally, and sleeping soundly, and desires to know how soon, and how well, I think he will be able to walk.—Ed.

seen some of the sorest arms after vaccination, and thinks it due to an erysipelatous epidemic, which now prevails to a great extent.

*Dr. Boardman* described his manner of vaccination; uses the scale reduced to a pulp, and rubs it in, after making crosses, not deep enough to draw much blood, if he could avoid it.

*Dr. Ring* related cases similar to those presented by Dr. Rochester, and spoke of the comparative value of the lymph, and of the virus as contained in the scale, and also of the protection afforded by vaccination.

*Dr. Gay* would make one observation. He was himself re-vaccinated a few years since without effect; subsequently was to be exposed to small pox by going to pest house; was now again re-vaccinated and it worked perfectly. The question now arises, would visiting the small pox hospital have anything to do with the operation of vaccination?

*Dr. Lockwood* remarked that he had recently, as an experiment, re-vaccinated some children that had been vaccinated six years previously, and had good scars, with virus, the product of re-vaccination, and to his surprise it worked finely, passing through all the stages of true vaccine disease.

*Dr. Rochester* thought it unsafe to use the crusts of re-vaccination, since in this way vaccination is liable to deteriorate; often has had good crusts, but rejects them as unfit for use. It might be well enough to make the experiment, but it was not safe to depend upon its protective influence.

*Dr. Boardman* said that the scar could not be depended upon as evidence of a protective influence of vaccination; would often get operation when the scar was well marked and perfect.

*Dr. Cronyn* inquired, if the operation of re-vaccination was evidence of impurity in the first, is not or may not the scale of re-vaccination be perfect?

*Dr. Rochester* referred to a report made to the Society by himself upon this subject, and published in the *Buffalo Medical Journal*, in which all these points had been considered. Working of re-vaccination was not always proof that the first was imperfect.

*Dr. Boardman* said, we often hear physicians and others, speak of vaccination running out, but we do not mean by that that the patient would have small pox if exposed; he might have varioloid, but never true, small pox.

*Prevailing Diseases.*—Drs. Rochester, Ring, Cronyn, Shaw, Boardman, Gay and Samo, reported the prevalence of erysipelas, scarlet fever, tonsilitis, diphtheria, etc., etc.

*Dr. Samo* related the case of a little boy he was attending, who was not very sick for the first few days, though somewhat feverish with loss of sleep and appetite. It was a few days ago covered with a papular eruption, and now covered by ptechial spots, appearing something like purpura.—Thinks it may be diphtheritic in character; has not however seen similar cases.

*Voted* to adjourn. J. F. MINER, Secretary.

## Figures and Tables

**Figure f1:**